# Supplementary material for: Alphavirus-Driven Interferon Gamma (IFNg) Expression Inhibits Tumor Growth in Orthotopic 4T1 Breast Cancer Model
Source: Vaccines (Basel). 2021 Oct 27;9(11):1247. doi: 10.3390/vaccines9111247 (PMC8620866; doi:10.3390/vaccines9111247)
Supplement: Supplementary file 1 [file vaccines-09-01247-s001.zip › vaccines-1334081-supplementary.pdf]

vaccines

MDPI

a.

b.

c.

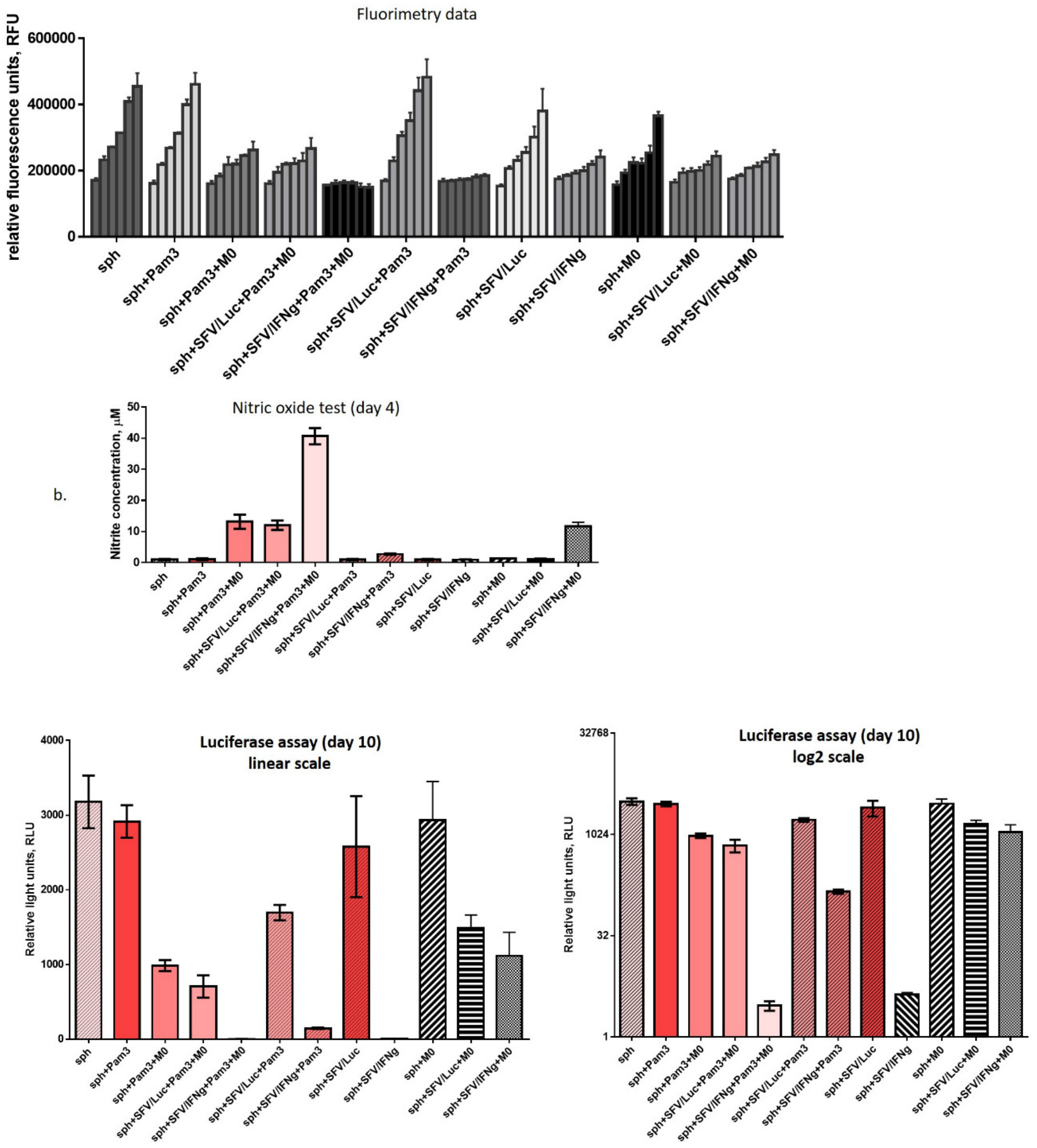

Figure S1. Analysis of 4T1/eGFP spheroids cultured in the presence of macrophages, Pam3 and SFV vectors. Single 4T1/eGFP spheroids (sph) were generated from 3000 cells (full name of the cell line is the 4T1-Fluc-Neo/eGFP-Puro, Imanis Life Sciences, USA) in 96-well ultralow attachment plates. The next day, the spheroids were infected with either SFV/IFNg or SFV/Luc (5 ×10^4 i.u./well) or incubated with PBS as the uninfected control. For infection, the virus solution was added to the pre-washed spheroids (PBS+Mg/Ca) incubated 1h and washed out as described in methods. The infected spheroids were incubated 18h in complete medium in a humidified 5% CO2 incubator at 37 °C. The next day after infection, BMDMs (3 × 10^4 cells/well) were added to the spheroids (+M0, day 0). In total, twelve combination groups (six spheroids in each group, n=6) were generated: sph – uninfected spheroids (PBS); sph+Pam3; sph+Pam3+M0;

sph+SFV/Luc+Pam3+M0; sph+SFV/IFNg+Pam3+M0; sph+SFV/Luc+Pam3; sph+SFV/IFNg+Pam3; sph+SFV/Luc; sph+SFV/IFNg; sph+M0; sph+SFV/Luc+M0; sph+SFV/IFNg+M0. Pam3 was added to respective groups together with/without macrophages to reach a final concentration 100ng/ml. **(a)** Fluorimetry data obtained by six measurements (six columns in each group) at days 0, 2, 4, 6, 8, 10. Each column represents the respective day of measurement (six columns for each group). **(b)** Nitric oxide analysis in the medium of each group at day 4. **(c)** Luciferase assay of cell lysates at day 10, according to Promega Luciferase assay kit (Catalog number: E1500): the spheroids were transferred into Eppendorf tubes, washed with PBS and resuspended with 30 µl of the lysis buffer, incubated 10 min on ice and centrifuged at 500 g to remove insoluble cell debris, then 20 µl of the cell lysate was mixed with 100 µl D-luciferin solution and used for luminometry by Luminoskan Ascent (Thermo Scientific, UK)The Luciferase is constitutively produced by 4T1/eGFP cells (4T1-Fluc-Neo/eGFP-Puro cells, see methods). The results of the assay were not influenced by the presence of SFV/Luc virus in infected cells, as by day 10, the 4T1 spheroids (without the luciferase expression) initially infected with SFV/Luc demonstrate no detectable expression of luciferase (not shown). Bars represent the mean value ± SD, n=6.

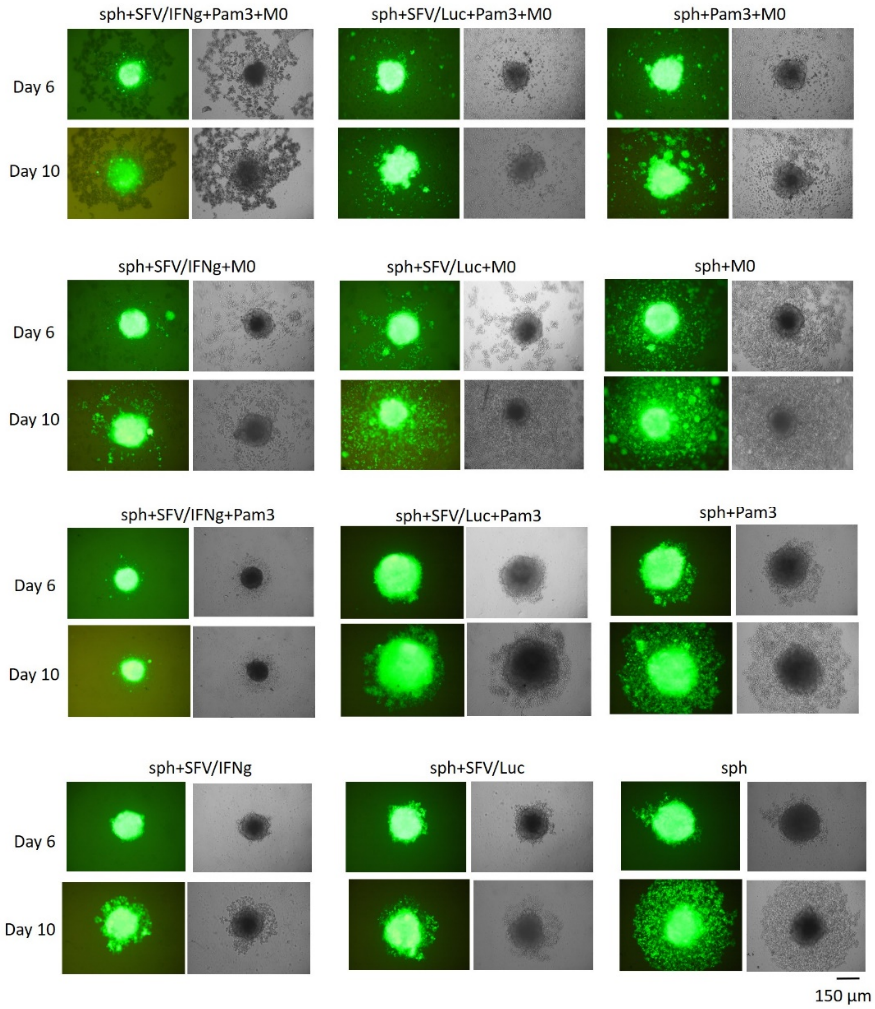

Figure S2. Microscopy of 4T1/eGFP spheroids cultured in the presence of macrophages, Pam3 and SFV vectors. Single 4T1/eGFP spheroids were generated from 3000 cells in 96-well ultralow attachment plates (as

described in methods). The next day, the spheroids were infected with either SFV/IFNg or SFV/Luc (5×10^4 i.u./well) or incubated with PBS as the uninfected control. The next day after infection, BMDMs (3×10^4 cells/well) were added to the spheroids (+M0, day 0). In total, twelve combination groups (six spheroids in each group, n=6) were prepared: sph – uninfected spheroids (PBS); sph+Pam3; sph+Pam3+M0; sph+SFV/Luc+Pam3+M0; sph+SFV/IFNg+Pam3+M0; sph+SFV/Luc+Pam3; sph+SFV/IFNg+Pam3; sph+SFV/Luc; sph+SFV/IFNg; sph+M0; sph+SFV/Luc+M0; sph+SFV/IFNg+M0. Representative images from each group at incubation day 6 and day 10 are shown: fluorescent microscopy image (on the left) and corresponding phase-contrast image (on the right). The microscopy was implemented by inverted contrasting microscope Leica DM-IL. One illustrative image per group is presented; the groups correspond to the groups presented in Figure S1.

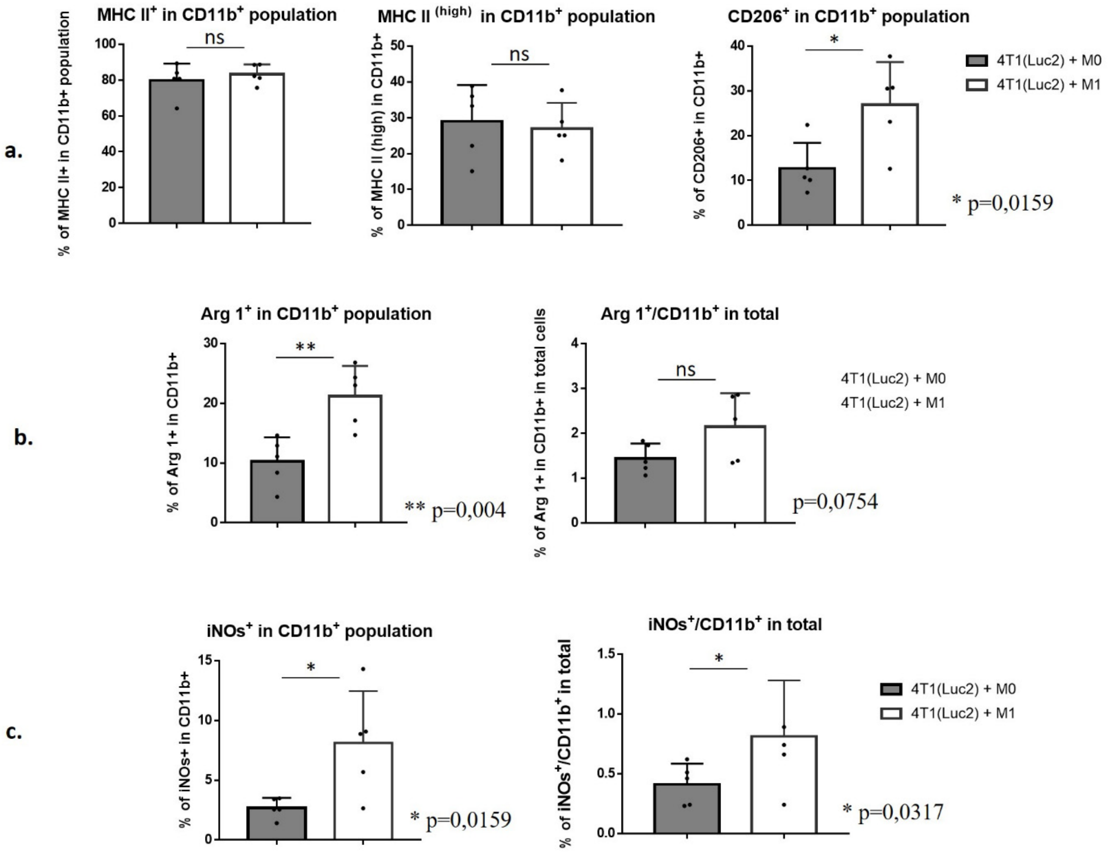

Figure S3. Flow cytometry analysis of immune cells isolated from tumours generated by implantation of 4T1(Luc2) cells premixed with M0 or M1 macrophages. For orthotopic implantation, 1×10^4 cells 4T1(Luc2) cells were mixed with either 2×10^4 M0 (4T1(Luc2)+M0) or 2×10^4 M1-like cells activated with vdIFNg/Pam3 (4T1(Luc2)+M1) in total volume of 50 µl. After 15 days, the mice were anaesthetized and humanely sacrificed, the tumours were homogenized and a single cell suspension was used for immunostaining (see Materials and methods for the details). Flow cytometry was performed to quantify (%) the immune cell populations: MHC II^+, MHC $II^{(high)}$, $CD206^+$ in $CD11b^+$ population; Arginase 1 (Arg 1^+) in $CD11b^+$ population, and in total isolated single cells; inducible NO synthase ($iNOs^+$) in $CD11b^+$ population, and in total isolated single cells. Bars represent the mean ± SD (n = 5); *p* values are indicated; ns – nonsignificant.

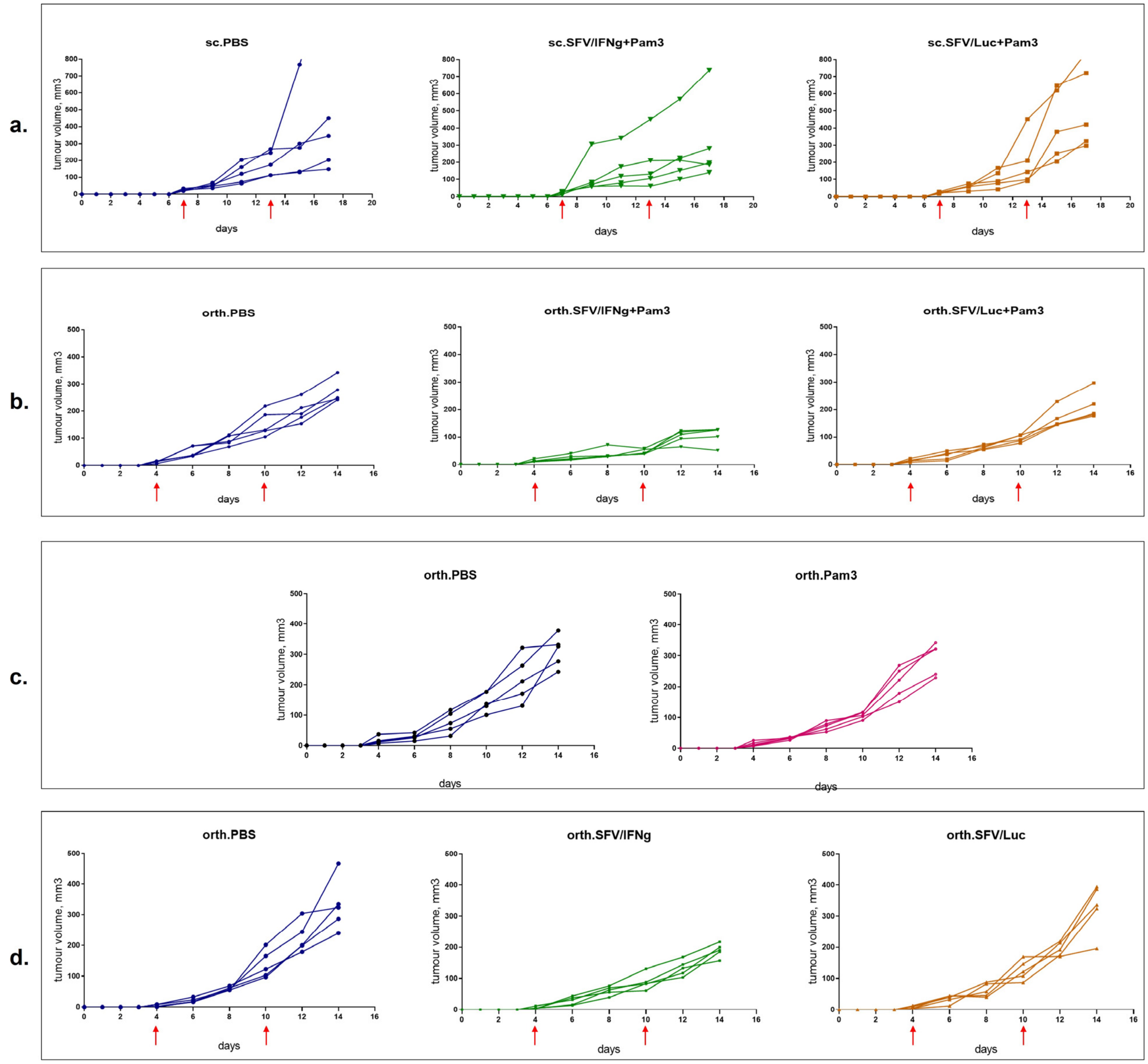

Figure S4. **The tumour growth curves for all individual animals treated by i.t. injection of SFV/IFNg virus and respective controls.** The 4T1 mouse breast tumours were established by subcutaneous (sc.), or orthotopic (orth.) injections of 2.5×10^5 or 1.25×10^5 4T1 cells, respectively. Mice received two i.t. injections of SFV vectors ($4{\times}10^7$ i.u./tumour) or PBS control. Time points when the treatment was received are marked with the red arrows. Volume of tumours was measured every two days. **(a)** Treatment of subcutaneous tumours with PBS, SFV/IFNg, and SFV/Luc. The next day after virus administration, mice received i.t. injections of Pam3 solution (10 µg/tumour after first virus administration and 15 µg/tumour after second virus administration). **(b)** Treatment of orthotopic tumours with PBS, SFV/IFNg, and SFV/Luc. The day after virus administration, mice received i.t. injections of Pam3 solution (10 µg/tumour after first virus administration and 15 µg/tumour after second virus administration). **(c)** Treatment of orthotopic tumours with Pam3 solution: first i.t. injection of 10 µg Pam3 at day 5; second i.t. injection of 15 µg Pam3 at day 11. **(d)** Treatment of orthotopic tumours with PBS, SFV/IFNg, and SFV/Luc, without the subsequent Pam3 injection.

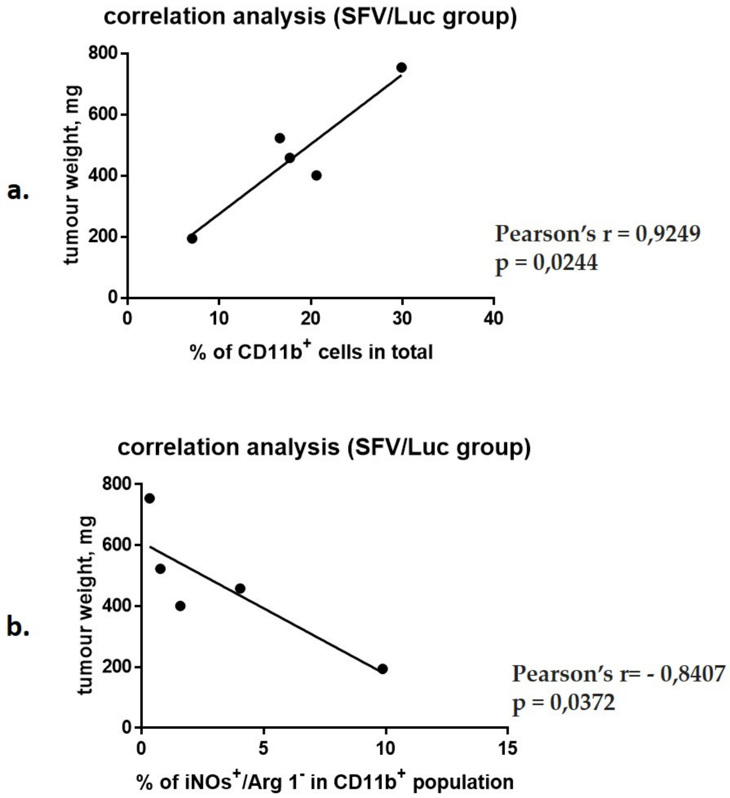

Figure S5. Pearson's **correlation analysis of tumours treated with SFV/Luc virus in orthotopic 4T1 model.** The 4T1 mouse breast tumours were established by orthotopic (orth.) injections of 1.25×10^5 4T1 cells. Mice received two i.t. injections of SFV/Luc vector (4×10^7 i.u./tumour), as described in methods. After 15 days, the mice were anaesthetized and humanely sacrificed, the tumours were resected, the weight was measured, then the tumours were homogenized and a single cell suspension was used for immunostaining (see Materials and methods for the details). Flow cytometry was performed to quantify (%) the immune cell populations. **(a)** Correlation analysis of the tumor weight versus the percent of $CD11b^+$ cells. **(b)** Correlation analysis of the tumor weight versus the percent of inducible NO synthase (iNOs) positive/arginase 1 (Arg 1) negative cells in CD11b+ population. r - Pearson's correlation coefficient. $p<0,05$ is considered statisticaly significant.

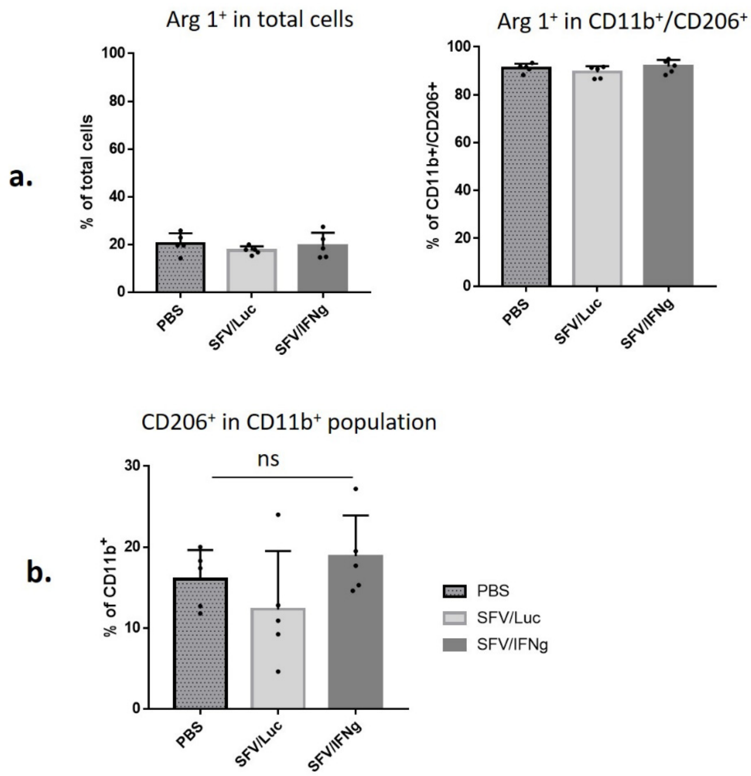

Figure S6. Flow cytometry analysis of immune cells isolated from tumours treated with SFV/IFNg, SFV/Luc viruses, or PBS in orthotopic 4T1 model. The 4T1 mouse breast tumours were established by orthotopic (orth.) injections of 1.25×10^5 4T1 cells. Mice received two i.t. injections of SFV/Luc, or SFV/IFNg vectors ($4{\times}10^7$ i.u./tumour), or PBS, as described in methods. After 15 days, the mice were anaesthetized and humanely sacrificed, the tumours were resected, homogenized and a single cell suspension was used for immunostaining (see Materials and methods for the details). Flow cytometry was performed to quantify (%) the immune cell populations: **(a)** Arginase 1 (Arg 1^+) in total isolated single cells and Arg 1^+ within double positive $CD11b^+/CD206^+$ cell population; **(b)** $CD206^+$ cells in $CD11b^+$ population. Bars represent the mean ± SD (n = 5); ns – nonsignificant (p>0,05).
